# A Preterm Case of Cow’s Milk Allergy Presenting with Recurrent Ascites Treated with Donor Breast Milk

**DOI:** 10.3390/ijerph18031187

**Published:** 2021-01-29

**Authors:** Ruka Nakasone, Kazumichi Fujioka, Shutaro Suga, Shinya Abe, Mariko Ashina, Kosuke Nishida, Motoichiro Sakurai, Katsumi Mizuno, Kandai Nozu, Kazumoto Iijima

**Affiliations:** 1 Department of Pediatrics, Graduate School of Medicine, Kobe University, 7-5-1 Kusunoki-cho, Chuo-ku, Kobe 650-0017, Japan; nakasone@med.kobe-u.ac.jp (R.N.); sugashu@med.kobe-u.ac.jp (S.S.); sabe@med.kobe-u.ac.jp (S.A.); marikoa@med.kobe-u.ac.jp (M.A.); nk1125@med.kobe-u.ac.jp (K.N.); nozu@med.kobe-u.ac.jp (K.N.); iijima@med.kobe-u.ac.jp (K.I.); 2 Department of Pediatrics, School of Medicine, Showa University, 1-5-8 Hatanodai, Shinagawa-ku, Tokyo 142-8666, Japan; kii0124@med.showa-u.ac.jp (M.S.); katsuorobi@med.showa-u.ac.jp (K.M.)

**Keywords:** breast feeding, birth weight, cow’s milk allergy, preterm infant, ascites, donated breast milk, newborn

## Abstract

We report a case of a preterm infant who developed cow’s milk allergy. This male infant presented with recurrent ascites and was successfully treated with donated breast milk. He was born at 24 weeks’ gestation with a birthweight of 506 g. From day 20, infant formula, soy protein-based formula, and casein-hydrolyzed formula were used due to insufficient maternal lactation. This resulted in abdominal distention, generalized edema, and recurrent ascites. We diagnosed him with cow’s milk allergy since these symptoms improved on exclusive breast milk feeding. No recurrence of symptoms occurred when donated breast milk was used in combination with the mother’s own milk. Ascites should be regarded as a clinical symptom of neonatal cow’s milk allergy. Donated breast milk may be effective in the treatment of the allergy if breastfeeding is not available.

## 1. Introduction

According to the European birth cohort study, the prevalence of cow’s milk allergy in infancy has been reported to be approximately 1% [1]. However, due to a diversity of symptoms and heterogeneity of diagnostic criteria, the diagnosis of cow’s milk allergy in infancy is difficult. Thus, the actual prevalence is estimated to be actually higher than that reported [2]. Incidence and clinical symptoms of cow’s milk allergy in preterm infants have not been fully elucidated [3]. Miyazawa et al., in a Japanese nationwide survey, reported that the incidence of clinically diagnosed neonatal cow’s milk allergy was 0.21% in all high-risk neonates and 0.35% in neonates weighing less than 1000 g [4]. They also reported that gastrointestinal symptoms, including diarrhea, bloody stool, vomiting, and abdominal distension were the main clinical manifestations of neonatal cow’s milk allergy [5].

It is widely accepted that cow’s milk allergy is much less frequent in breastfed infants [6]. In a single-center retrospective study, Morita et al. reported that 83% of infants with cow’s milk allergy were fed cow’s milk-based formula [7]. Although breastfeeding has not been shown to reduce cow’s milk protein allergy as reported in a recent systematic review [8], breastfeeding is regarded as having many benefits for infants and is a good option to manage neonatal cow’s milk allergy [6,8]. However, breastfeeding of all high-risk infants in neonatal intensive care units is not easy. In addition, preterm infants have a lower success rate of exclusive breastfeeding than do term infants [9]. Therefore, the American Academy of Pediatrics (AAP) recommends using pasteurized donor breast milk (DBM) for the nutritional management of very low birthweight infants (weight < 1500 g), in case of insufficient milk supply from their own mothers despite adequate support [10]. To provide DBM appropriately, breast milk banks have been established in Europe since 1909 [11]. Currently, there are 24 milk banks in North America (20 in the United States and 4 in Canada) that pasteurize milk as part of the Human Milk Banking Association of North America reported in 2017 [12]. Moreover, 226 milk banks in 28 European countries as part of the European Milk Bank Association have been reported in 2019 [11]. In Japan, despite the establishment of the Human Milk Bank Association in 2017, only one breast milk bank has recently provided pasteurized DBM [13]. 

Breastfeeding offers the best treatment strategy for cow’s milk allergy, with highly hydrolyzed formula or amino acid-based formula being alternatives depending on the severity of allergy [6,14,15]. Here, we report a case of cow’s milk allergy presenting with recurrent abdominal distension and ascites, which was successfully treated with milk bank-provided DBM.

## 2. Case History

A male infant was born to a 23-year-old woman via emergency cesarean section at 25 weeks and 6 days gestation due to non-reassuring fetal status. The infant’s birthweight was 506 g, with an APGAR score of 2 at 1 min and 8 at 5 min. He was immediately intubated and treated with artificial pulmonary surfactant for respiratory distress syndrome. After admission, his cardiopulmonary status was stabilized by routine management. We started enteral feeding on day 4 with breastmilk, reaching full feeding (>100 mL/kg/day) on day 15. Preterm formula (LW94^®^, Meiji Co., Ltd., Tokyo, Japan) was started on day 20 due to maternal lactation insufficiency. In addition, human milk fortified with HMS-1^TM^ and HMS-2^TM^ (Morinaga Milk Industry Co. Ltd., Tokyo, Japan) was added on days 20 and 27, respectively, to increase calorie intake. Subsequently, we changed the preterm formula to infant formula (Lebens Milk HAIHAI, Asahi Group Holdings, Ltd., Tokyo, Japan) to manage significant hyperglycemia on day 29.

**Figure 1:**
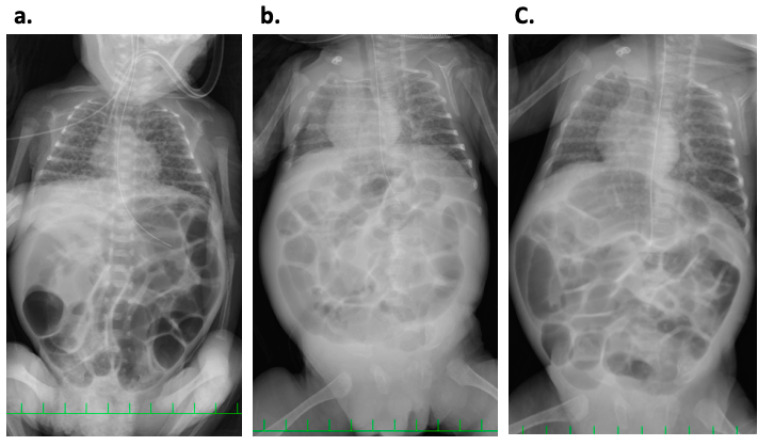
Abdominal X-ray of the case. Massive bowel dilatation was noted on (**a**) day 36, (**b**) day 85, and (**c**) day 98.

Since then, there have been three episodes of marked abdominal distension associated with respiratory deterioration, requiring discontinuation of enteral feeding. Abdominal distension recurred when using infant formula on days 36 and 46 (Figure 1a), and even after using a soy protein-based formula (ELENATL^®^ P, EA Pharma Co., Ltd., Tokyo, Japan) on day 55, with no exclusive breast feeding. Based on the reproducibility of symptoms, we suspected cow’s milk allergy to the infant formula and/or soy protein-based formula. Since lactation of the mother was insufficient, we tried to use a casein-hydrolyzed formula (new MA-1, Morinaga Milk Industry Company, Ltd., Tokyo, Japan) on day 66. In addition, we re-started the human milk fortifier HMS-1^TM^ to improve bodyweight gain. 

**Figure 2:**
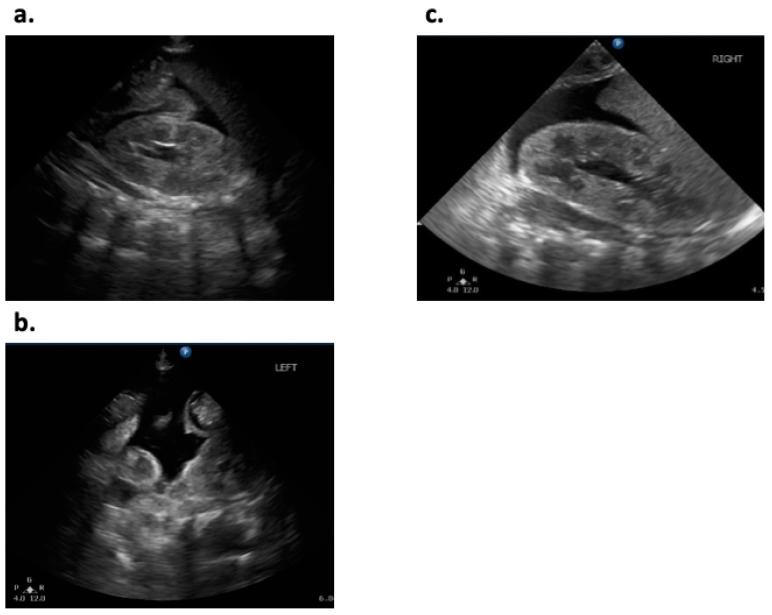
Abdominal ultrasonography of the case. Ascites was noted on (**a**,**b**) day 85 and (**c**) day 98.

On day 85, abdominal distension recurred, and was associated with generalized edema, loose stool, and lethargy. Blood tests revealed significant hypoproteinemia (Total protein; 3.2 g/dL, Albumin; 2.2 g/dL) with no increase in markers of infection (white blood cell; 6100/µL, CRP; 0.02 mg/dL). Abdominal X-ray revealed marked dilation of intestinal gas and elevated diaphragm. Abdominal ultrasonography showed ascites (Figure 1b and Figure 2a,b). 

Thus, we stopped enteral feeding and intravenously administered 25% albumin; subsequently, the abdominal distension, edema, and ascites improved. After confirming resolution of ascites, we restarted enteral feeding exclusively with the mother’s milk on day 87. Subsequently, we added casein-hydrolyzed formula on day 94. However, we had to stop feeding on day 98 due to recurrence of abdominal distension, general edema, and ascites (Figure 1c and Figure 2c). Based on these events, we diagnosed the infant with cow’s milk allergy to the casein-hydrolyzed formula, in addition to the infant formula and/or soy protein-based formula. Since we could not obtain enough of the mother’s milk, in addition to unavailability of amino acid-based formula in our institute, we decided to use donated breast milk (DBM) provided by the breast milk bank after obtaining approval from the Kobe University Clinical Ethics Committee and written parental informed consent. We used the DBM together with the mother’s milk from day 107, and the patient showed good body weight gain without any gastrointestinal symptoms (Table 1).

**Figure 3:**
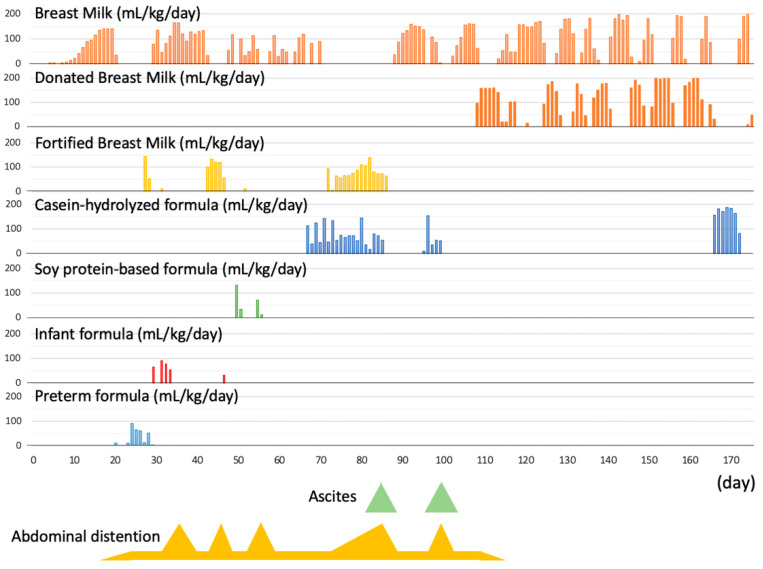
Clinical course of the case. Abdominal distention and ascites occurred when the infant was fed with milk other than breast milk from either the infant’s mother’s milk or donated breast milk.

A drug-induced lymphocyte stimulation test performed on day 153 was negative for casein-hydrolyzed formula (149%), but positive for soy protein-based formula (233%). Based on this result, we restarted casein-hydrolyzed formula together with the mother’s milk on day 164. Since no gastrointestinal symptoms recurred, the infant was discharged with this feeding method on day 173 (Figure 3). An allergen-specific lymphocyte stimulation test performed on day 170 was negative for human-α-lactalbumin and bovine-κ-casein, but positive for bovine-lactoferrin.

## 3. Discussion

In the present case, recurrent ascites with abdominal distension and respiratory deterioration were noted. However, typical symptoms of cow’s milk allergy such as vomiting and bloody stool were not observed [1]. This infant was also intolerant to casein-hydrolyzed formula and soy protein-based formula, which are recommended as treatment options for cow’s milk allergy. Therefore, the infant required treatment with DBM provided by the milk bank.

For assessment of the clinical symptoms of cow’s milk allergy, Vandenplas et al. created the Cow’s Milk-related Symptom Score (CoMiSS^TM^), which includes the following: crying, regurgitation, stools, skin symptoms, and respiratory symptoms [16]. This score showed excellent inter-rater variability between pediatricians and parents [17], and was shown to be useful for predicting positive results in the challenge test [18]. Therefore, it is now widely used in assessing symptoms of Cow’s milk allergy in infants and young children [19,20]. However, symptoms of neonatal cow’s milk allergy have not been fully elucidated. According to a nationwide survey on neonatal cow’s milk allergy in Japan, almost 90% of cases had gastrointestinal symptoms, which included vomiting (44.1%), bloody stool (40.5%), abdominal distension (28.8%), diarrhea (23.4%), poor sucking (19.8%), and increased gastric residual (9.1%). However, there have been no reports of ascites [5]. In addition, a single-center study in the neonatal intensive care unit reported that gastrointestinal symptoms were the most common symptoms of neonatal cow’s milk allergy, including vomiting (71%), bloody stool (63%), less vigorous sucking (21%), abdominal distention (8%), and gastric hemorrhage (4%). However, no ascites was reported [7]. We considered protein-losing gastroenteropathy caused by an immune reaction as the mechanism for ascites in this case, which is widely noticed in gastrointestinal food allergies in children [21].

To the best of our knowledge, there have been no reports on the use of DBM for the treatment of neonatal cow’s milk allergy. Although there have been concerns about the risk of transmitting infectious pathogens through DBM, Blackshaw et al. in their literature review revealed that the majority of cases of infant morbidity were associated with unpasteurized milk (not sourced through milk banks) and expressed with inappropriate technique. In addition, powdered infant formula use has been reported to be associated with a direct intrinsic and extrinsic pathogen transmission risk [22]. Regarding the risk of necrotizing enterocolitis (NEC), Adhisivam et al. reported in their randomized controlled trial that the incidence of NEC in infants receiving pasteurized DBM was not increased even in the groups in which commercially available fortifiers were added (2.5%), compared to those that were unfortified (7.5%) [23]. This might suggest the safety of DBM used in combination with fortification. Another benefit is that DBM is also known to contain significantly higher nutritional antioxidants than those in formula milk, although not as high as those of the infant’s mother’s milk [24]. Thus, we believe DBM provided by the breast milk bank was a safe and reasonable treatment option for this case.

DBM is mainly used in preterm infants, infants after gastrointestinal tract operations, infants whose mothers cannot breastfeed due to illness or hospitalization, infants with metabolic diseases, and infants immediately after birth before their own mother’s milk becomes available [25]. In addition, according to a 2015 Korean report, 93.1% of DBM users were preterm infants [26]. In Chinese reports, recipients consisted of preterm infants (*n*  =  2990, 63.9%), and those due to feeding intolerance (*n*  =  711, 15.2%), maternal illness (*n*  =  345, 7.4%), serious infection (*n*  =  314, 6.7%), NEC (*n*  =  244, 5.2%), post-surgery (*n*  =  38, 0.8%), and others (*n*  =  36, 0.8%), in descending order [27]. Similarly, in Taiwan, the order was: prematurity (65.4%), malabsorption (7.6%), feeding intolerance (7.2%), maternal illness (5.1%), and post-surgery (4.6%) [28]. Our patient had allergic symptoms not only to infant formula, but also to casein-hydrolyzed formula and soy protein-based formula used as a treatment, and only breast milk was well tolerated. Casein-hydrolyzed formula and soy protein-based formula are generally used in the treatment of cow’s milk allergy. However, they can cause allergic reactions [29]. Regarding the infant formulae that were used, the preterm formula contained both casein and whey, the casein-hydrolyzed formula contained casein but not whey, and the soy protein-based formula did not contain either casein or whey. Interestingly, in recent studies, IgE and IgG cross-reactive allergens and epitopes were found between alpha1-casein and soybean protein [30]. We speculated that these cross-reactivities could also have been present in our case, and a whey-based hydrolyzed formula could have resolved this problem; however, it was not adopted at our facility. In addition, it has been reported that cow’s milk allergies are less likely to occur in exclusively breastfed infants [6], and thus our patient might not have been affected if exclusive breastfeeding was achieved. Since the patient was an extremely low birth weight (< 1000 g) preterm infant, we tried to promote breastfeeding. However, insufficient milk letdown was due to inadequate lactation counselling because of the mother’s distant residence as well as the ban on hospital visits occasioned by the COVID-19 pandemic. DBM is recommended for preterm infants or very low birthweight infants whose own mother’s milk volume is not enough or cannot be used [12,31]. Based on the findings of this case, we suggest that DBM provided by milk banks should be considered as an effective alternative in infants with Cow’s milk allergy.

## 4. Conclusions

Ascites in preterm infants can occur as part of the clinical symptoms of neonatal cow’s milk allergy, for which DBM may be an effective treatment in neonates who are intolerant to casein-hydrolyzed and soy protein-based formulae.

## Figures and Tables

**Table 1:** Anthropometric parameter over time.

Days	0	30	60	90	120	150	173	197	253
Bodyweight (g)	506	802	946	1222	1486	1954	2490	3221	4713
Height (cm)	N/A	31.4	35.2	36.4	38.4	43.0	44.0	49.4	56.9
Head circumference (cm)	N/A	24.0	26.0	28.0	30.2	32.4	33.0	35.0	38.2
Chest circumference (cm)	N/A	20.0	24.0	23.2	26.4	28.4	29.0	36.0	39.6

N/A; data not available.

## Data Availability

The data presented in this study are available in the article.
